# Brain-Specific Ultrastructure of Capillary Endothelial Glycocalyx and Its Possible Contribution for Blood Brain Barrier

**DOI:** 10.1038/s41598-018-35976-2

**Published:** 2018-11-30

**Authors:** Yoshiaki Ando, Hideshi Okada, Genzou Takemura, Kodai Suzuki, Chihiro Takada, Hiroyuki Tomita, Ryogen Zaikokuji, Yasuaki Hotta, Nagisa Miyazaki, Hirohisa Yano, Isamu Muraki, Ayumi Kuroda, Hirotsugu Fukuda, Yuki Kawasaki, Haruka Okamoto, Tomonori Kawaguchi, Takatomo Watanabe, Tomoaki Doi, Takahiro Yoshida, Hiroaki Ushikoshi, Shozo Yoshida, Shinji Ogura

**Affiliations:** 1 Department of Emergency and Disaster Medicine, Gifu University Graduate School of Medicine, Gifu, Japan; 2 Department of Internal Medicine, Asahi University School of Dentistry, Mizuho, Japan; 3 Department of Tumor Pathology, Gifu University Graduate School of Medicine, Gifu, Japan; 4 Laboratory of Molecular Biology, Department of Biofunctional Analysis, Gifu Pharmaceutical University, Gifu, Japan; 5 Research Institute for Biotechnology, Asahi University School of Dentistry, Mizuho, Japan; 6 Department of Clinical Laboratory, Gifu University Hospital, Gifu, Japan

## Abstract

Endothelial glycocalyx coats healthy vascular endothelium and plays an important role in vascular homeostasis. Although cerebral capillaries are categorized as continuous, as are those in the heart and lung, they likely have specific features related to their function in the blood brain barrier. To test that idea, brains, hearts and lungs from C57BL6 mice were processed with lanthanum-containing alkaline fixative, which preserves the structure of glycocalyx, and examined using scanning and transmission electron microscopy. We found that endothelial glycocalyx is present over the entire luminal surface of cerebral capillaries. The percent area physically covered by glycocalyx within the lumen of cerebral capillaries was 40.1 ± 4.5%, which is significantly more than in cardiac and pulmonary capillaries (15.1 ± 3.7% and 3.7 ± 0.3%, respectively). Upon lipopolysaccharide-induced vascular injury, the endothelial glycocalyx was reduced within cerebral capillaries, but substantial amounts remained. By contrast, cardiac and pulmonary capillaries became nearly devoid of glycocalyx. These findings suggest the denser structure of glycocalyx in the brain is associated with endothelial protection and may be an important component of the blood brain barrier.

## Introduction

The brain contains various systems for maintaining the homeostasis necessary for proper neurological function. Among these systems, the blood-brain barrier (BBB) plays a central role. The components of the BBB include (1) cerebral capillaries that lack fenestration and show extremely low rates of transcellular vascular transport; (2) tight junctions between the endothelial cells and tightly regulated intercellular transport^1–5^, and (3) pericytes and dendrites of astrocyte that surround the basement membrane to control substance influx and efflux^6–8^. In addition, the endothelial glycocalyx, which coats healthy vascular endothelium, confers a negative electric charge to the surface of endothelial cells, thereby forming an electrical barrier^9^. Notably, degradation of the glycocalyx reportedly leads to blood-brain barrier dysfunction^10^. It is therefore thought that endothelial glycocalyx is crucial for maintaining brain homeostasis.

Sugar-protein glycocalyx overlays the vascular endothelium^11–13^ and plays key roles in microvascular and endothelial physiology, including regulation of endothelial permeability, leukocyte adhesion, and nitric oxide production^14–20^. We previously reported that the morphology of glycocalyx varies among the different types of capillaries: in continuous capillaries, the endothelial glycocalyx exhibits moss- or broccoli-like features and is present over the entire luminal surface of the endothelial cells; in fenestrated capillaries, the glycocalyx appears to nearly occlude the endothelial pores; and in sinusoidal capillaries, the glycocalyx does not occlude the open fenestrations and is thinner than in continuous or fenestrated capillaries^21^.

Although cerebral capillaries are categorized as continuous, like the capillaries in the heart and lung, the vascular endothelial structure greatly differs from those in other organs due to its functional specificity. However, there have been few reports directly examining the morphology of glycocalyx in cerebral capillaries. The purpose of the present study was to identify the ultrastructure of endothelial glycocalyx on cerebral capillaries and compare that structure to those in the cardiac and pulmonary capillaries. We hypothesized that the structure of endothelial glycocalyx in the cerebral capillaries is distinct from that in other organs.

## Results

### Ultrastructure of Cerebral Capillary Glycocalyx

**Figure 1:**
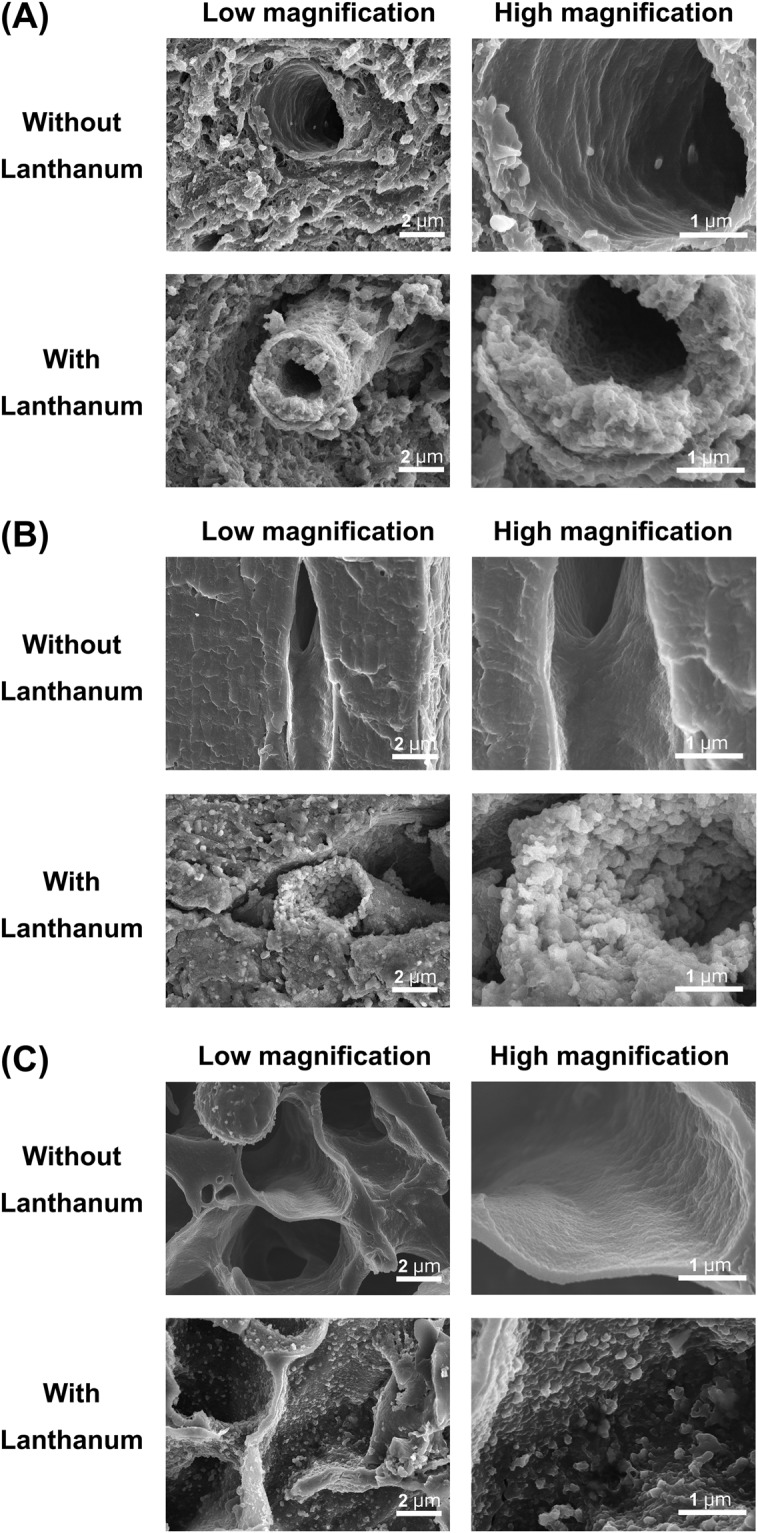
Scanning electron micrographs showing the ultrastructure of continuous capillaries in the (**A**) brain, (**B**) heart and (**C**) lung. Upper panels: without lanthanum nitrate staining. Lower panels: with lanthanum nitrate staining for visualization of endothelial glycocalyx. Panels on the right are expanded views of those on the left. Continuous capillaries have a continuous basement membrane, and the endothelial glycocalyx can be seen on the surface of the vascular endothelial cells.

**Figure 2:**
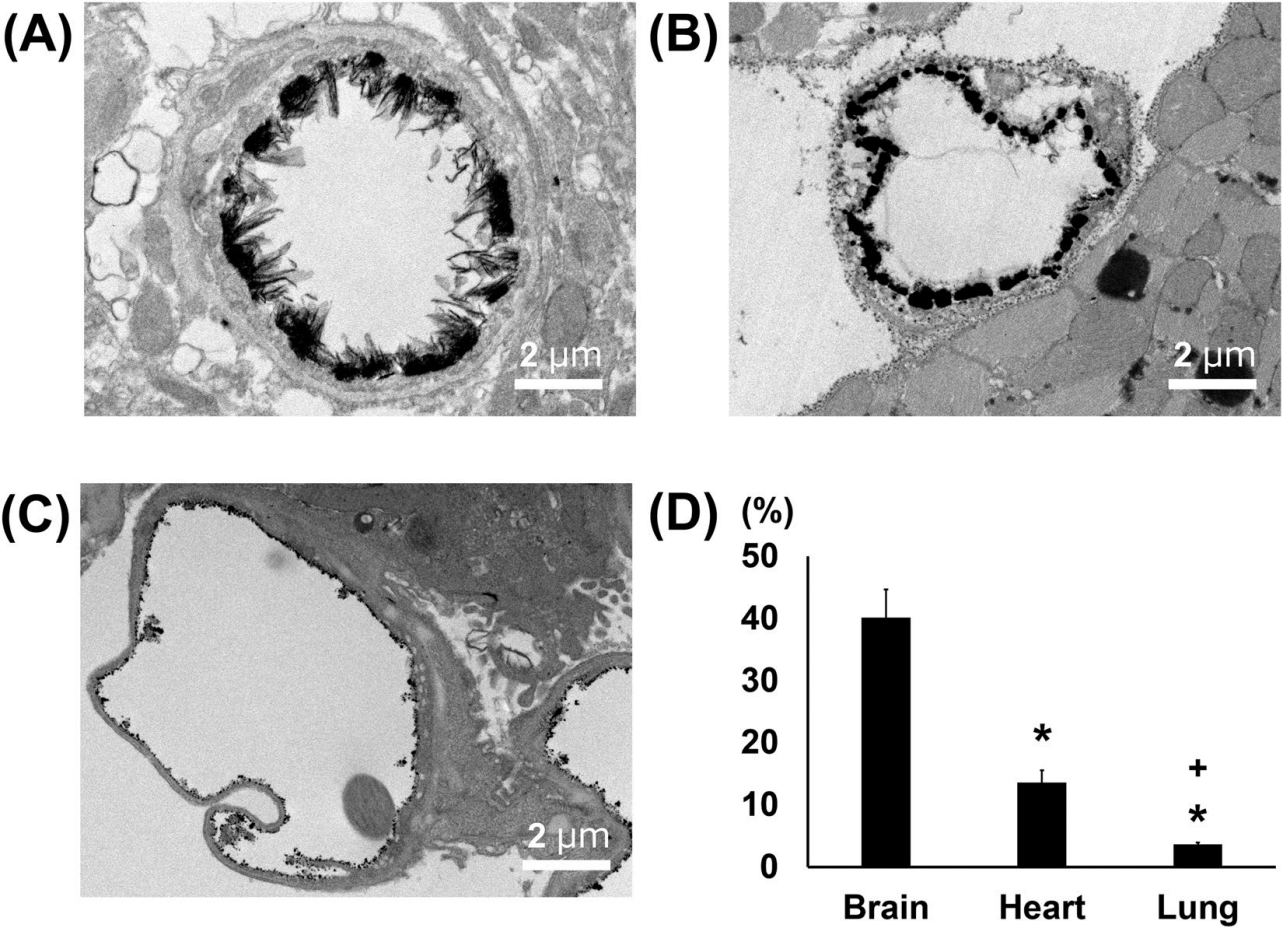
Transmission electron microscopic analysis of continuous capillaries. (**A**–**C**) Cerebral (**A**), cardiac (**B**) and pulmonary (**C**) capillaries with lanthanum nitrate staining. The endothelial glycocalyx covers the surface of vascular endothelial cells. (**D**) Percent area covered by endothelial glycocalyx in capillaries of brain, heart and lung. Bars indicate means ± SE. *p < 0.05 vs brain.

Capillaries in the brain are classified as continuous. To confirm the surface structure of normal cerebral capillaries, we first performed an ultrastructural analysis using scanning and transmission electron microscopy (SEM and TEM, respectively) without lanthanum nitrate staining. Standard SEM examination of the luminal side of these capillaries showed an uninterrupted endothelium (Fig. 1A). In addition, the dendrites of astrocytes could be seen surrounding the cerebral capillaries **(**Supplementary Fig. 1**)**. Cerebral endothelial glycocalyx visualized using lanthanum nitrate staining revealed moss- or broccoli-like structures on the endothelial cells (Fig. 1A). These structures were entirely invisible in the absence of lanthanum nitrate staining. Endothelial glycocalyx was also visualized using lanthanum nitrate staining in the heart and lung, where the capillaries are also characterized as continuous (Fig. 1B,C). However, the structures of the endothelial glycocalyx greatly differed among brain, heart and lung; it appeared densest in the brain and denser in the heart than in the lung. For quantitative analysis, we observed endothelial glycocalyx under TEM (Fig. 2A–C). The percentage of endothelial surface covered by glycocalyx in the capillaries was 40.1 ± 4.5%, 15.1 ± 3.7% and 3.7 ± 0.3%, while the average length of the endothelial glycocalyx was 301.0 ± 111.8 nm, 135.5 ± 59.7 nm, and 65.4 ± 28.4 nm, in the brain, heart and lung, respectively (Fig. 2D, Supplementary Fig. 2).

### Ultrastructure of Glycocalyx Injuries in Continuous Capillaries

**Figure 3:**
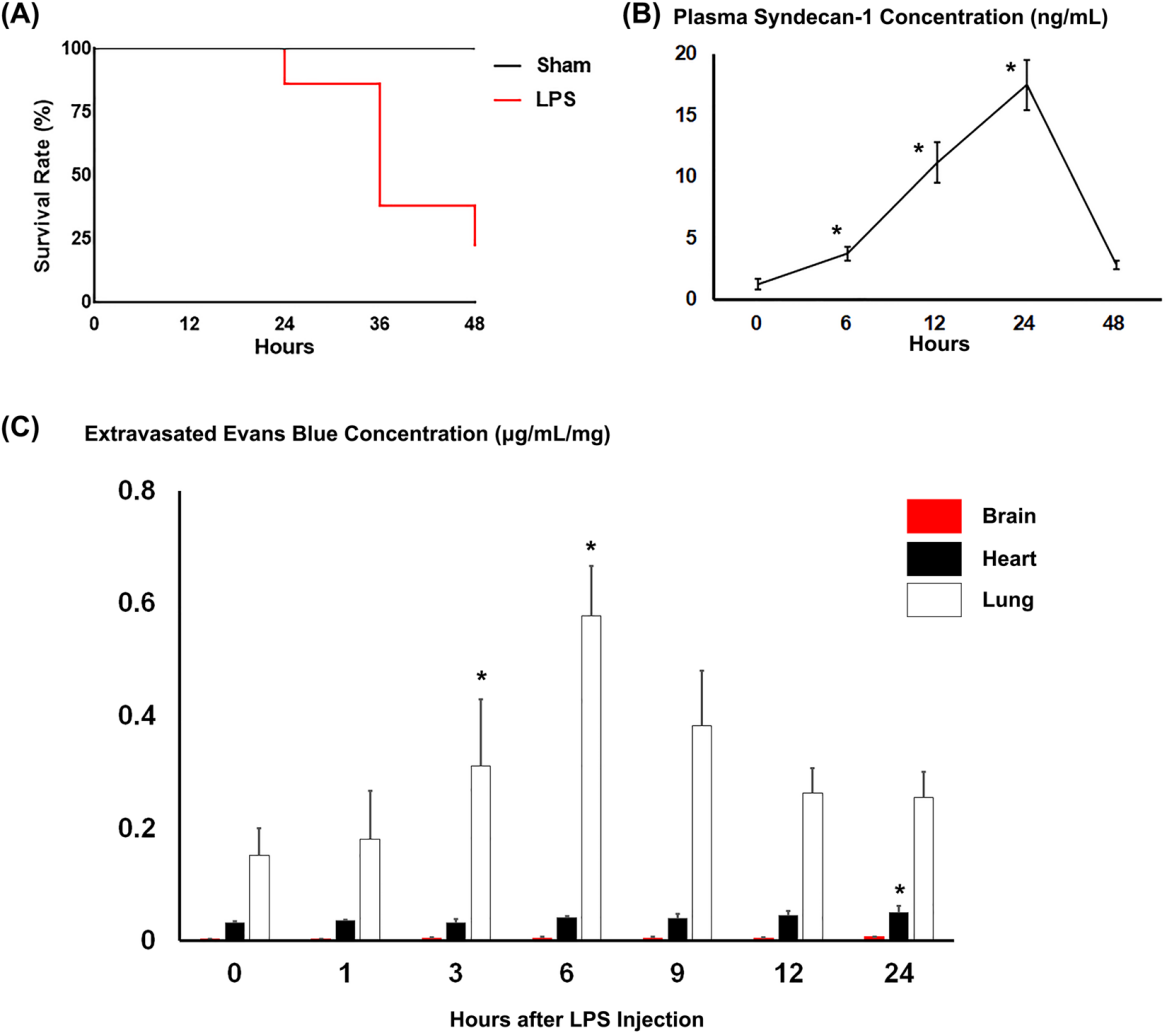
Characterization of LPS-injected mice. (**A**) Survival after LPS administration. Forty-eight h after LPS administration, the survival rate of mice was 22% (11/50 injected mice). (**B**) Plasma syndecan-1 concentrations at the indicated times after LPS administration. *p < 0.05 vs. before LPS administration. (**C**) Extravasated Evans blue levels in brain, heart and lung at the indicated times after LPS administration. Levels were normalized to organ weight. Bars indicate means ± SE. *p < 0.05 vs before LPS administration in each organ.

Ten-week-old C57BL6 male mice were intraperitoneally injected with lipopolysaccharide (LPS) and sacrificed up to 48 h after LPS injection to see the extent of glycocalyx injury. Only 11 of the 50 injected mice survived for the 48 h (Fig. 3A). Plasma syndecan-1 levels, a marker of glycocalyx injury, were significantly increased 6, 12 and 24 h after LPS administration However, there was no significant difference between syndecan-1 levels before and 48 h after LPS administration. (Fig. 3B).

To quantitatively analyze blood vessel permeability, we measured extravasation of Evans blue (Fig. 3C). In pulmonary capillaries, the amount of Evans blue extravasation was significantly higher 3 h after LPS injection than in sham mice and reached a peak 6 h after LPS administration. By contrast, extravasation of Evans blue was more modest in the heart and was nearly absent in the brain (Fig. 3C). After sham treatment, the extravascular Evans blue concentration in mouse brain was 0.0032 ± 0.0010, 0.0029 ± 0.0004 and 0.0026 ± 0.0006 µg/mL/mg 6, 12 and 24 h after PBS injection. This did not significantly differ from the levels before LPS injection (0.0028 ± 0.0007 µg/mL/mg).

**Figure 4:**
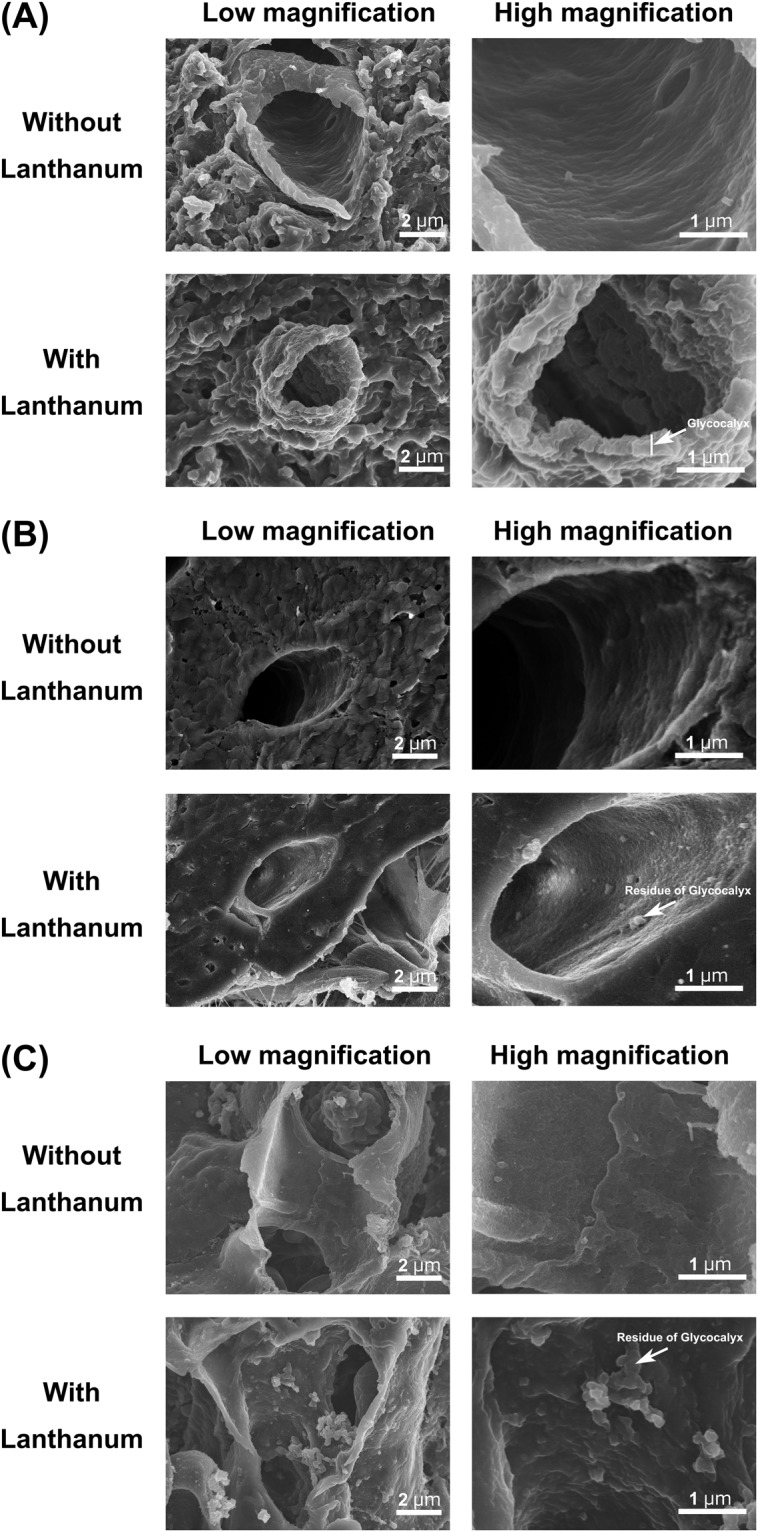
Scanning electron micrographs showing the ultrastructure of continuous capillaries 48 h after LPS administration in the (**A**) brain, (**B**) heart and (**C**) lung. Upper panels: without lanthanum nitrate staining, Lower panels: with lanthanum nitrate staining for visualization of endothelial glycocalyx. Right panels are expanded views of the left panels in each figure. After LPS administration, the endothelial glycocalyx is degraded on the surface of the vascular endothelium in the heart and lung, but the glycocalyx is maintained in the brain.

**Figure 5:**
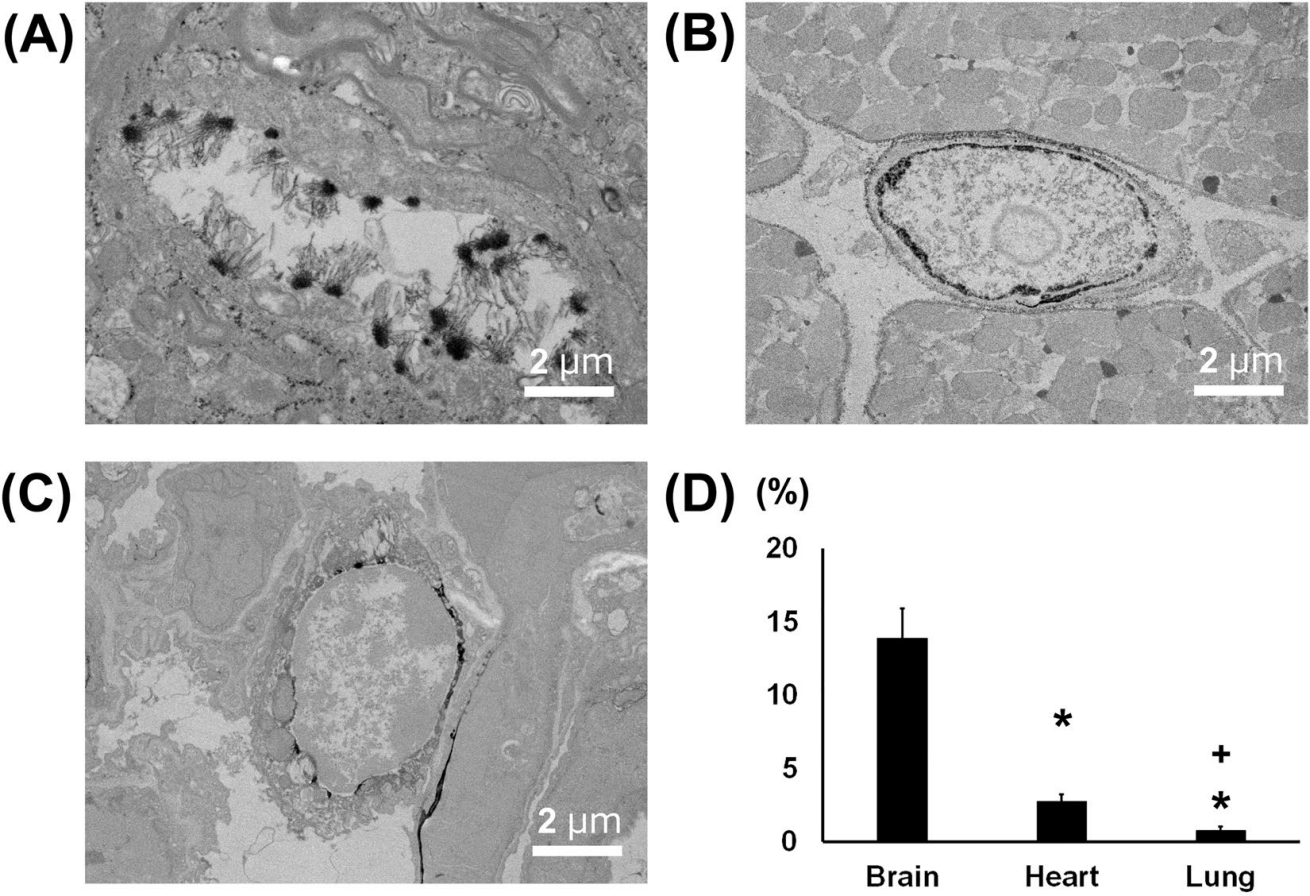
Transmission electron microscopic analysis of continuous capillaries 48 h after LPS administration. (**A**–**C**) Cerebral (**A**), cardiac (**B**) and pulmonary (**C**) capillaries with lanthanum nitrate staining. The endothelial glycocalyx has degraded on the surface of vascular endothelial cells in the heart and lung, but not in the brain. (**D**) Percent area covered by endothelial glycocalyx in capillaries of brain, heart and lung. Bars indicate means ± SE. *p < 0.05 vs brain.

We next performed an ultrastructural analysis to assess structural changes in the endothelial glycocalyx induced by injury. After LPS administration, standard SEM examination revealed that the luminal surface of brain capillary was not injured and almost normal (Fig. 4A). Although the glycocalyx in cerebral capillaries was also injured after LPS, it was still present on the vessel surface and covered the endothelial cells (Fig. 4A). By contrast, in cardiac and pulmonary capillaries, the glycocalyx had mostly degraded on the lumenal surface of the capillaries to form debris after LPS administration, and the endothelial cells were directly exposed to the blood cells (Fig. 4B,C). The percent area of endothelial surface covered by glycocalyx in pulmonary, cardiac and cerebral continuous capillaries was significantly lower following LPS injection than in sham mice (Fig. 5). However, there were large differences the amount of glycocalyx remaining on the endothelial surface within the three organs. In cerebral capillaries, the percent area covered by the remaining glycocalyx was 13.6 ± 2.0%, whereas in the cardiac and pulmonary capillaries, it was only 2.8 ± 0.2% and 0.8 ± 0.2%, respectively.

## Discussion

Endothelial cell structure is specific for each organ and forms at least three types of capillaries: continuous, fenestrated and sinusoidal^22–24^. We previously used capillaries from the heart (continuous type), kidney (fenestrated type), and liver (sinusoidal type) to show that the structure of the endothelial glycocalyx differs among the three capillary types^21^. In the present study, we show that the structure of endothelial glycocalyx differs among capillaries in the brain, heart and lung, even though all are classified as continuous.

Previous investigations suggested that glycocalyx can act as a mechanosensor of fluid shear stress^15,25,26^. Consistent with that idea, fluid shear stress on endothelial cells increases levels of synthetic glycosaminoglycan, which is one component of endothelial glycocalyx^27–29^.

Earlier studies revealed that the thickness of the glycocalyx differed depending on the vascular lumen diameter, which ranges from 2–3 μm in small arteries to 4.5 μm in the carotid arteries^30,31^. On the other hand, another study reported that the glycocalyx in muscle capillaries form a 0.5-μm-thick layer that covers the surface of the endothelial cells^32^.

The present study showed that the cerebral capillary endothelial glycocalyx is denser than that in the heart, despite both organs being perfused under the same high-pressure system. Moreover, the thicker endothelial glycocalyx in brain may contribute to BBB function.

The BBB, which is specific to the brain, strictly regulates transport of metabolites between the blood and brain substrate. For example, a tight junction protein on cerebrovascular endothelial cells functions specifically to regulate vascular permeability and matrix metalloprotease activity^33^. Sepsis-associated encephalopathy is a diffuse functional brain disorder that occurs as a result of a systemic inflammatory reaction to infection and differs from encephalitis caused by invasion of the central nervous system by a microbe^34,35^. Although the underlying mechanism remains unknown, contributing to vascular endothelial cell dysfunction and BBB injury in these cases are reportedly LPS and inflammatory cytokines^34^. In the present study, the glycocalyx on cerebral endothelium was thinned by LPS administration, but substantial amounts persisted, and the endothelium was not injured. Indeed, 24 h after LPS administration there was no significant change in extravascular Evans blue levels. Although the injured brain vessel showed only 13% coverage, that is comparable to the level in normal cardiac capillaries. We therefore suggest that the remaining glycocalyx was sufficient to maintain the appropriate level of vascular permeability. In addition, unlike Evans blue bound to albumin, free Evans blue would not be restricted by the glycocalyx. In the present study, it is not clear that the injected free Evans blue was all bound to albumin. Because the accumulation of a tracer such as Evans blue is determined as much by the number of perfused vessels as by the vessels’ permeability, it is difficult to conclude that large changes in perfusion did not compromise the measurement of Evans blue accumulation without data on the vascular response to LPS. In cardiac and pulmonary capillaries, by contrast, LPS administration causes endothelial surfaces in capillaries to be exposed directly to the lumen, as the endothelial glycocalyx is largely degraded. However, even cerebral endothelial glycocalyx peels off in more severe cases of sepsis, leading to injury of the capillary endothelium. In other words, sepsis that causes cerebral endothelial injury is extremely serious. In fact, the mortality rate among sepsis patients with central nervous system involvement is higher than among patients without it^36–39^.

The present study has several limitations. Because lanthanum has the capacity to bind not only to glycocalyx but also calcium binding sites, it has been used as a calcium probe in several biological systems^40^. It is therefore hard to say that the lanthanum staining technique is specific for glycocalyx. In addition, lanthanum nitrate staining for glycocalyx visualization may itself influence the glycocalyx structure. The present study revealed differences in the extent to which the glycocalyx is preserved in different organs. However, it is not clear whether this represents the undisturbed state of the glycocalyx or whether the structure is preserved when the glycocalyx is stained.

The purpose of using LPS in the present study was to assess the ultrastructural alteration of endothelial glycocalyx in different organ capillaries. Because we previously established a severe septic vasculitis model using 20 mg/kg LPS administration^21,41^, we used that protocol again in the present study. In this model, severe vasculitis was induced in mice through intraperitoneal administration of LPS after 16 h of starvation. We therefore chose to inject Evans Blue 16 h prior to injecting LPS at the same time starvation was initiated. This protocol, by itself, may have affected the results of the study.

Finally, our findings do not reveal a direct correlation between changes in the glycocalyx and capillary function because we did not investigate the integrity of the tight junctions or alterations in vesicular trafficking after LPS treatment.

In summary, the present study shows that the form of the endothelial glycocalyx in the brain differs from that in the heart and lung, despite capillaries in all three organs being classified as continuous. It appears, the denser glycocalyx in the brain may provide added endothelial protection there and may function as a component of the BBB.

## Materials and Methods

### Animals

This study conforms to the Guide for the Care and Use of Laboratory Animals and was approved by the Institutional Animal Research Committee of Gifu University (Gifu, Japan). Ten-week-old C57BL6 mice were used. Blood was collected from the ophthalmic artery before sacrifice, after which brain, heart and lung specimens were collected. All quantified data were from surviving mice. In a preliminary study, we confirmed that the shape of the endothelial glycocalyx does not significantly differ between male and female mice. We used male mice in this experiment, as in previous reports^21,41^.

### Electron Microscopy

To detect endothelial glycocalyx using electron microscopy, mice were anesthetized and perfused with lanthanum-containing alkaline solution^21^. Before perfusion, an incision was made in the right atrial appendage, and the abdominal aorta was ligated with a silk suture for better perfusion of the heart, lung and brain. A perfusion pump was used for injection at a steady pace of 1 ml/min. Sample preparation for SEM and TEM was as described previously^21^.

### Vascular Injury Model and an *in vivo* Assay for Blood Vessel Permeability

Ten-week-old male mice were first intraperitoneally administered a sterile solution of Evans blue in PBS (WAKO, Japan, 100 µg/kg) and then starved for 16 h. The mice were then intraperitoneally administered LPS (20 mg/kg; Sigma Aldrich) and sacrificed 0, 1, 3, 6, 12, 24 and 48 h after the LPS administration (n = 6 at each time point). Prior to sacrifice, the mice were perfused with PBS containing 2 mmol/l EDTA to washout the Evans blue solution from vessel lumens. After sacrifice, the hearts, lungs and brains were collected, and tissues samples were placed in tubes and dried at 65 °C in an oven to eliminate variation in water content. Thereafter, 500 µl of formamide was added to each tube, and the samples were incubated at 60 °C for an additional 24 h to extract the Evans blue from tissue. Following the Evans blue extraction, the absorbance of the solution at 610 nm was measured, and the amount of extravasated Evans blue per mg tissue was calculated.

### Measurement of Syndecan-1 in the Plasma

Following LPS administration to mice, plasma concentrations of syndecan-1 were measured (n = 6) using an ELISA (Diaclone, Besancon Cedex, France; 860.090.192).

### Quantitative Assessment of Endothelial Glycocalyx Area

Quantitative assessment of the endothelial glycocalyx occupation area within capillary lumens was performed on 6 randomly chosen capillary vessels in TEM images using ImageJ software (National Institutes of Health, Bethesda, MD, USA). In each vessel, the vascular lumenal area was measured, and the area of glycocalyx was measured using image thresholding **(**Supplementary Fig. 3**)**. The glycocalyx occupation was then calculated from these values.

### Quantitative Assessment of Endothelial Glycocalyx Length

Quantitative assessments of endothelial glycocalyx length were performed on 6 randomly chosen capillary vessels in TEM images using ImageJ software. The length of the endothelial glycocalyx was measured at 10 randomly chosen points in each vessel.

### Statistical analysis

Values are shown as means ± SE. Survival was analyzed using the Kaplan-Meier method with the log-rank Cox-Mantel method. The significance of differences was evaluated using *t*-tests. Values of *P* < 0.05 were considered significant.

## Electronic supplementary material

Supplementary Figures
