# Supplementary material for: Brain-Specific Ultrastructure of Capillary Endothelial Glycocalyx and Its Possible Contribution for Blood Brain Barrier
Source: Sci Rep. 2018 Nov 30;8:17523. doi: 10.1038/s41598-018-35976-2 (PMC6269538; doi:10.1038/s41598-018-35976-2)
Supplement: Supplementary file 1 — Supplementary Figures [file 41598_2018_35976_MOESM1_ESM.docx]

**Brain-Specific Ultrastructure of Capillary Endothelial Glycocalyx and Its Possible Contribution for Blood Brain Barrier**

Yoshiaki Ando^1+^, Hideshi Okada M.D., Ph.D.^1+*^, Genzou Takemura M.D., Ph.D.^2^, Kodai Suzuki M.D., Ph.D.^1^, Chihiro Takada B.S.^1^, Hiroyuki Tomita M.D., Ph.D.^3^, Ryogen Zaikokuji B.S.^4^, Yasuaki Hotta Ph.D.^5^, Nagisa Miyazaki M.D., Ph.D.^2^, Hirohisa Yano^1^, Isamu Muraki^1^, Ayumi Kuroda^1^, Hirotsugu Fukuda^1^, Yuki Kawasaki^1^, Haruka Okamoto M.D.^1^, Tomonori Kawaguchi M.D., Ph.D.^1^, Takatomo Watanabe M.D., Ph.D.^6^, Tomoaki Doi M.D., Ph.D.^1^, Takahiro Yoshida M.D.^1^, Hiroaki Ushikoshi M.D., Ph.D.^1^, Shozo Yoshida M.D., Ph.D.^1^, Shinji Ogura M.D., Ph.D.^1^

^1^Department of Emergency and Disaster Medicine, Gifu University Graduate School of Medicine, Gifu, Japan; ^2^Department of Internal Medicine, Asahi University School of Dentistry, Mizuho, Japan; ^3^Department of Tumor Pathology, Gifu University Graduate School of Medicine, Gifu, Japan; ^4^Laboratory of Molecular Biology, Department of Biofunctional Analysis, Gifu Pharmaceutical University, Gifu, Japan; ^5^Research Institute for Biotechnology, Asahi University School of Dentistry, Mizuho, Japan; and ^5^Department of Clinical Laboratory, Gifu University Hospital, Gifu, Japan

+ These authors contributed equally.

**Address for Correspondence:**

Hideshi Okada, M.D., Ph.D.

Department of Emergency and Disaster Medicine,

Gifu University Graduate School of Medicine,

1-1 Yanagido, Gifu 501-1194, Japan

Phone; +81-58-230-6448; Fax; +81-58-230-6451

E-mail: [hideshi@gifu-u.ac.jp](mailto:hideshi@gifu-u.ac.jp)

**Keywords:** Endothelial Glycocalyx; Ultrastructure; Vascular Permeability

Total word count: 3820 words, Abstract word count: 177 words; No. of figures: 5, No. of tables: 0





**Supplementary Figure 1**

Left panel: Image of a brain capillary with lanthanum nitrate staining (the same as in Figure 1A). Right panel: Expanded view of the white square in the left panel. A white arrow indicates an astrocyte on the cerebral capillary; black arrows point to the astrocyte foot processes.


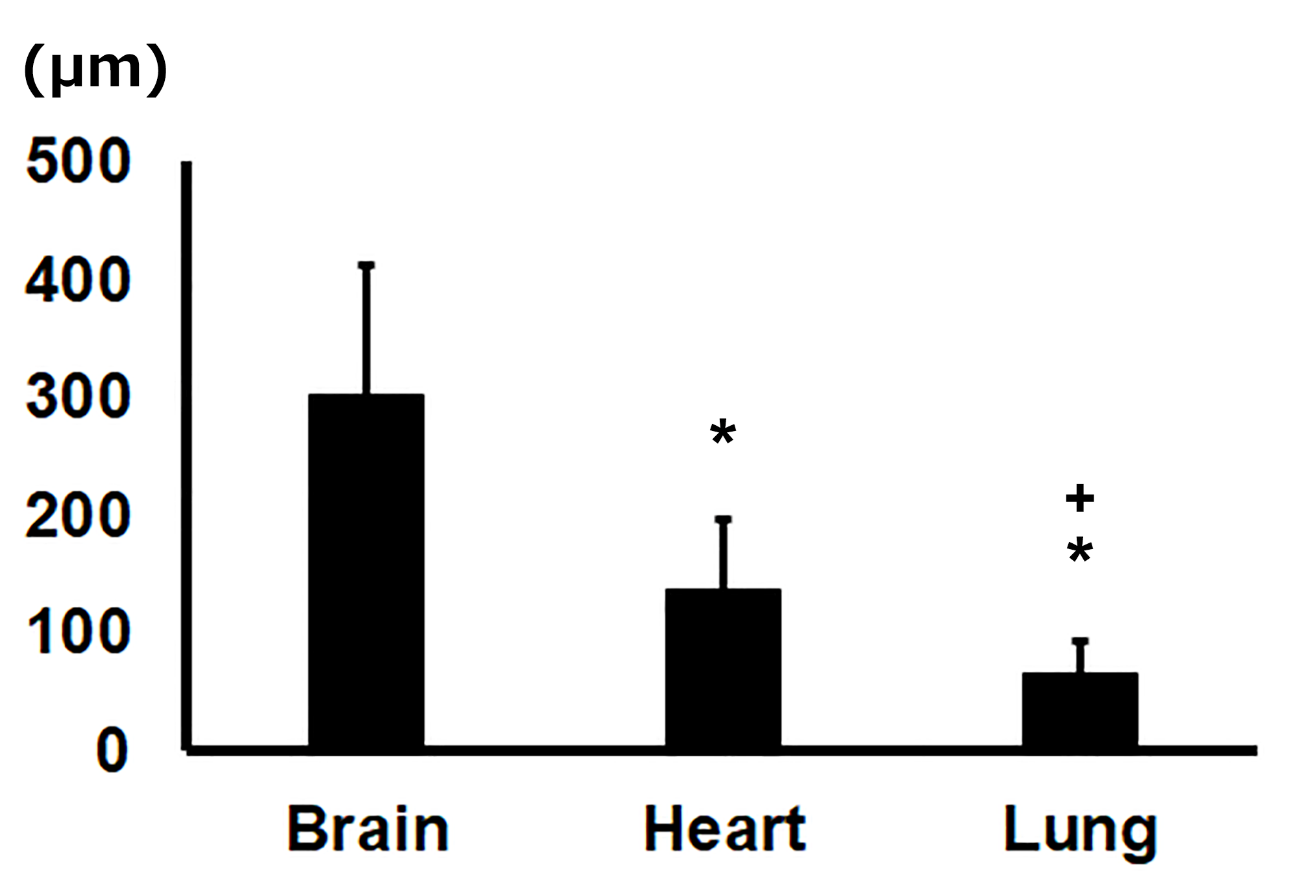


**Supplementary Figure 2**

Lengths of endothelial glycocalyx in the brain, heart and lung in sham and LPS-injected mice. * *p* < 0.05 vs brain, + *p* < 0.05 vs heart


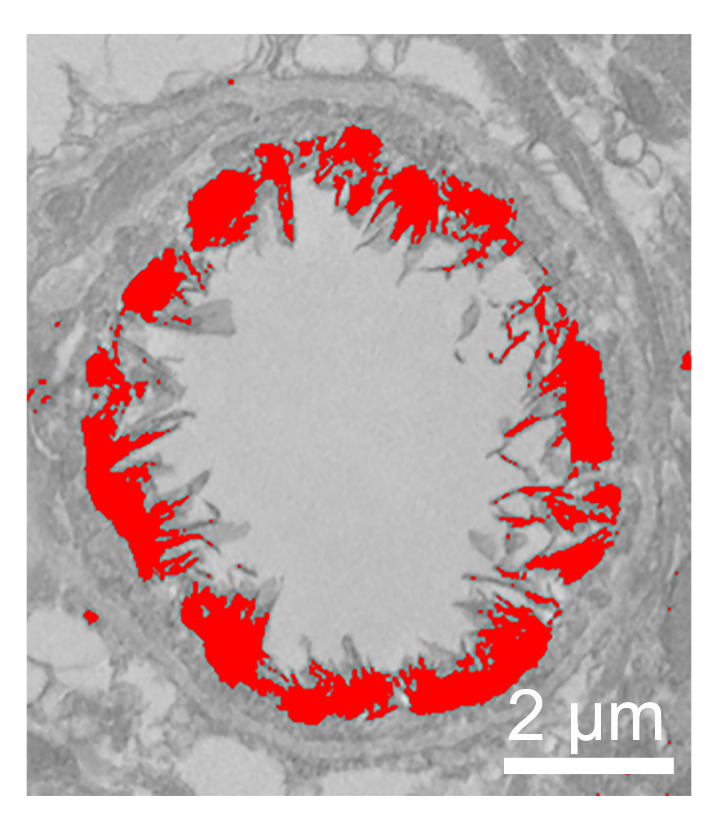


**Supplementary Figure 3**

Measurement of glycocalyx area. The red area represents endothelial glycocalyx determined using image thresholding with ImageJ software.
